# Comprehensive genetic diagnosis of Japanese patients with severe proteinuria

**DOI:** 10.1038/s41598-019-57149-5

**Published:** 2020-01-14

**Authors:** China Nagano, Tomohiko Yamamura, Tomoko Horinouchi, Yuya Aoto, Shinya Ishiko, Nana Sakakibara, Yuko Shima, Koichi Nakanishi, Hiroaki Nagase, Kazumoto Iijima, Kandai Nozu

**Affiliations:** 1 Department of Pediatrics, Kobe University Graduate School of Medicine, 7-5-2 Kusunoki-cho, Chuo-ku, Kobe, Hyogo 650-0017 Japan; 2 Department of Pediatrics, Wakayama Medical University, 811-1 Kimiidera, Wakayama, Wakayama, 641-8509 Japan; 3 Department of Child Health and Welfare (Pediatrics), Graduate School of Medicine, University of the Ryukyus, 207 Uehara, Nishihara-cho, Okinawa 903-0125 Japan

**Keywords:** Diseases, Medical research, Nephrology, Risk factors, Signs and symptoms

## Abstract

Numerous disease-causing gene mutations have been identified in proteinuric diseases, such as nephrotic syndrome and glomerulosclerosis. This report describes the results of comprehensive genetic diagnosis of Japanese patients with severe proteinuria. In addition, the report describes the clinical characteristics of patients with monogenic disease-causing mutations. We conducted comprehensive gene screening of patients who had either congenital nephrotic syndrome, infantile nephrotic syndrome, steroid-resistant nephrotic syndrome, or focal segmental glomerular sclerosis. Using targeted next-generation sequencing, 60 podocyte-related genes were screened in 230 unrelated patients with proteinuria. A retrospective review of clinical data was conducted for these patients. We detected monogenic disease-causing mutations in 30% (69 of 230) of patients among 19 of the screened genes. Common genes with disease-causing mutations were *WT1* (25%), *NPHS1* (12%), *INF2* (12%), *TRPC6* (10%), and *LAMB2* (9%). With various immunosuppressive or renoprotective therapies, remission of proteinuria in patients with unknown causative mutations was observed in 26% of patients, whereas only 5% of patients with monogenic disease-causing mutations exhibited complete remission. We assessed the genetic backgrounds of Japanese patients with severe proteinuria. The proportion of patients with gene defects was similar to that of other reports, but the disease-causing gene mutation frequency was considerably different.

## Introduction

Idiopathic nephrotic syndrome has an estimated incidence of approximately 2 to 6.5 per 100,000 children per year, depending on ethnic background^1,2^. Steroid-resistant nephrotic syndrome (SRNS) occurs in approximately 10%–20% of children with nephrotic syndrome, and is associated with an increased risk of complications due to persistent proteinuria and therapeutic drug side effects. Most patients with SRNS initially exhibit the histological pattern of focal segmental glomerulosclerosis (FSGS), which is the primary glomerular aetiology of end-stage renal disease (ESRD) in children^3,4^. Congenital nephrotic syndrome (CNS), which develops at 0–3 months of age, and infantile nephrotic syndrome (INS), which develops at 4–12 months of age, are most commonly associated with gene mutations that encode the structural and regulatory proteins of the glomerular filtration barrier^5^.

Whole-exome NGS sequencing has revealed new disease-causing gene mutations associated with SRNS. Recently, more than 50 podocyte-related gene mutations have been identified in monogenic forms of CNS/INS/SRNS/FSGS^6,7^. Genetic aetiologies were identified in 29.5% of patients with SRNS before 25 years of age in a Western population^8^ and in 28.3% of such patients in Chinese cohort^9^. The proportion of patients in whom disease-causing gene mutations could be detected decreased with increasing age at the onset of nephrotic syndrome in previous studies^10,11^. A genetic diagnosis may enable clinicians to begin early management and treatment, such as the discontinuation of immunosuppressant therapy due to its strong side effects, and the initiation of renoprotective drugs. In addition, disease-causing mutations in some genes encoding enzymes of the coenzyme Q10 (CoQ10) pathway can be treated by supplementation with CoQ10^12,13^. Finally, mutation detection analyses can facilitate the prediction of post-transplant recurrence of nephrotic syndrome^7,14^.

Studies of Western and Chinese cohorts of paediatric SRNS patients showed that the results of genetic analyses vary among ethnicities^8,9^. Following the advent of NGS, the discovery of proteinuric disease-causing gene mutations in patients has grown rapidly, although the exact incidence remains unclear. Until now, there have been no large-scale mutation screening studies of Japanese patients with nephrotic syndrome.

In this study, we used targeted NGS for simultaneous sequencing of 60 podocyte-related genes and aimed to clarify the clinical characteristics of Japanese patients with one of the following diseases: CNS, INS, SRNS, FSGS, or asymptomatic proteinuria with likely genetic disease.

## Results

### Mutations

We collected samples from facilities throughout Japan (Supplementary Fig. S1). A total of 230 unrelated patients (132 men/boys and 98 women/girls) were included. Their median age at disease onset was 3 years (range, 1 day to 65 years). Of these 230 patients, 10 were diagnosed with CNS, 14 were diagnosed with INS, 101 were diagnosed with SRNS, and 105 were diagnosed with FSGS or asymptomatic proteinuria. We detected disease-causing gene mutations in 69 of 230 unrelated patients (30%). The coverage depths of detected genes are shown in Supplementary Table S1. One of the 69 patients was from a consanguineous family, whereas 15 patients had a positive family history of proteinuria, and 15 patients had a positive family history of renal failure (Table 1). In the 25 patients harbouring autosomal recessive disease-causing mutations, four homozygous and 21 compound heterozygous mutations were found (Table 2).

**Table 1:** Clinical features of 69 Japanese patients with proteinuria for whom disease-causing mutations were identified.

Patient	Gene	Sex	Age at onset(y)	Category*	ESRD(y)	Histopathologic diagnosis	Family history**	Extra-renal symptom	Age at gene analysis(y)	eGFR (ml/min/1.73 m^2^)
Neph7	*WT1* (NM_024426.4)	F	0.33	2	0.5	DMS	—	—	0.75	5
Neph15	*WT1*	F	10	4	—	MGA	—	—	16	114.4
Neph33	*WT1*	F	3	4	—	MGA	—	—	9	113.5
Neph48	*WT1*	M	0.5	4	—	FSGS	—	micropenis, cryptorchidism	2	75.82
Neph52	*WT1*	F	6	3	—	FSGS	—	—	9	84.8
Neph90	*WT1*	M	3	3	12	FSGS	—	—	12	11.9
Neph92	*WT1*	F	0	1	0	—	—	—	0	14
Neph95	*WT1*	M	3	4	11	FSGS	—	—	30	33.9
Neph107	*WT1*	F	0.17	2	0.17	—	—	—	0.17	10.24
Neph132	*WT1*	F	0.5	2	0.5	sclerosis	—	—	1	3
Neph136	*WT1*	F	6	4	—	MGA	—	—	17	83.4
Neph137	*WT1*	F	2	3	—	Mesangial proliferative glomerulonephritis	—	—	5	100
Neph154	*WT1*	M	3	3	—	FSGS	—	cryptorchidism,hypospadias	14	21
Neph171	*WT1*	M	9	3	—	FSGS	—	—	29	25.8
Neph185	*WT1*	M	0.5	2	2	DMS	—	—	20	71.1
Neph197	*WT1*	F	3	4	7	FSGS	—	—	7	12.5
Neph208	*WT1*	F	0.42	2	0.42	—	—	—	0.5	18.76
Neph27	*NPHS1* (NM_004646.3)	M	0	1	—	—	—	—	0.08	34.1
Neph69	*NPHS1*	M	0.08	1	—	—	—	—	0.67	32.1
Neph91	*NPHS1*	F	8	3	—	MGA	—	—	20	244.3
Neph104	*NPHS1*	F	0	1	—	—	1	—	0.08	—
Neph106	*NPHS1*	F	0	1	—	—	—	—	0.17	55.5
Neph113	*NPHS1*	M	6	3	—	MGA	—	—	32	165.4
Neph192	*NPHS1*	M	0.75	3	—	MGA	—	—	2	92.3
Neph203	*NPHS1*	F	7	3	—	C1q nephropathy	1	—	10	195
Neph59	*INF2* (NM_022489.3)	F	13	4	—	Focal glomerular obsolescent	2	—	25	113
Neph76	*INF2*	M	10	4	25	FSGS	2	—	17	7.2
Neph134	*INF2*	M	9	4	—	FSGS	—	—	15	96.6
Neph155	*INF2*	M	11	4	14	FSGS	—	Charcot-Marie-Tooth disease	15	8.4
Neph177	*INF2*	M	30	4	—	FSGS	2	—	32	63
Neph198	*INF2*	F	13	4	—	MGA	—	—	16	115.2
Neph202	*INF2*	M	31	4	—	FSGS	2	—	39	12.6
Neph227	*INF2*	M	7	3	13	FSGS	—	—	22	53.2
Neph5	*TRPC6* (NM_004621.5)	F	3	4	6	FSGS	—	—	14	92.4
Neph24	*TRPC6*	F	4	3	4	FSGS	—	—	4	91.6
Neph39	*TRPC6*	F	3	3	7	C1q nephropathy	—	—	7	8.16
Neph139	*TRPC6*	F	2	4	—	FSGS	—	—	2	94.8
Neph149	*TRPC6*	M	7	3	—	MGA	—	—	8	95.17
Neph176	*TRPC6*	M	14	4	—	FSGS	2	—	14	140.13
Neph234	*TRPC6*	F	7	4	—	FSGS	2	—	7	119.8
Neph4	*LAMB2* (NM_002292.3)	M	0.33	2	—	FSGS	—	—	4	121
Neph23	*LAMB2*	F	0	1	0.25	Diffuse glomerular obsolescence	—	—	1	ESRD
Neph58	*LAMB2*	M	0	1	0	—	—	microcoria	0	4.1
Neph87	*LAMB2*	M	0.17	1	0.5	DMS	—	retinal detachment	10	68.9
Neph89	*LAMB2*	M	2	3	—	sclerosis	—	—	2	93.8
Neph133	*LAMB2*	F	0	1	0.08	—	—	choroiditis, chorioretinal atrophy	6	89.9
Neph36	*ADCK4* (NM_024876.3)	F	6	4	—	FSGS	—	—	8	89.7
Neph56	*ADCK4*	F	3	4	—	FSGS	—	—	4	111.2
Neph160	*ADCK4*	M	3	4	—	FSGS	—	—	5	60.8
Neph225	*ADCK4*	F	9	4	—	FSGS	1,2	—	11	90.9
Neph19	*NUP107* (NM_020401.2)	M	1.67	3	2.3	FSGS	—	—	5	6.2
Neph66	*NUP107*	F	3	4	—	—	—	—	3	129.4
Neph147	*NUP107*	M	7	4	—	FSGS	—	—	8	105.5
Neph68	*LMX1B* (NM_002316.3)	M	3	3	—	—	—	microcephaly	5	167.61
Npeh77	*LMX1B*	F	3	3	—	FSGS	2	—	19	109
Neph37	*ACTN4* (NM_004924.4)	M	6	4	13	FSGS	—	—	12	66.7
Neph146	*ACTN4*	M	8	3	—	FSGS	—	—	11	126
Neph129	*PAX2* (NM_003987.3)	M	3	4	—	FSGS	1	—	10	106.3
Neph230	*PAX2*	M	8	4	—	FSGS	—	—	8	47.3
Neph178	*COL4A5* (NM_000495.4)	F	8	3	—	FSGS	2	—	54	20.6
Neph204	*COL4A5*	F	1	4	—	non—IgA nephropathy	2	—	17	93.3
Neph97	*COQ6* (NM_182476.2)	M	0.75	2	—	—	—	—	0.92	83
Neph79	*FAT1* (NM_005245.3)	M	3	4	—	MGA	—	—	5	141.8
Neph189	*PLCE1* (NM_016341.3)	M	1	3	12	FSGS	1	—	21	70.16
Neph10	*SMARCAL1* (NM_014140.3)	M	7	3	—	FSGS	—	—	8	64.1
Neph143	*TTC21B* (NM_024753.4)	M	3	4	—	FSGS	—	situs inversus	4	57.1
Neph216	*MYH9* (NM_002473.5)	M	3	4	—	FSGS	—	—	8	100.1
Neph224	*CUBN* (NM_001081.3)	M	3	4	—	MGA	—	—	3	112
Neph236	*LAMA5* (NM_005560)	M	0.25	1	1	—	2	—	13	36.4

*1: Congenital nephrotic syndrome, 2: Infantile nephrotic syndrome, 3: Steroid-resistant nephrotic syndrome, 4: Focal segmental glomerular sclerosis or asymptomatic proteinuria.

**1: positive family history of proteinuria, 2: positive family history of renal failure.

Abbreviations: DMS, diffuse mesangial sclerosis; ESRD, end-stage renal disease; FSGS, focal segmental glomerular sclerosis; MGA, minor glomerular abnormality.

**Table 2:** Mutation genotypes of 69 Japanese patients with proteinuria for whom disease-causing mutations were identified.

Patient	Gene	genome	amino acids	Mode of Inheritance	Origin of variant	HGMD	reference allele read depth	alternative allele read depth	dbSNP	Japanese frequency
Neph7	*WT1* (NM_024426.4)	c.1384 C > T	p.Arg462Trp	AD	de novo	reported	16	12	rs121907900	No data(ND)
Neph15	*WT1*	c.1432 + 4 C > T		AD	Not done (ND)	reported	15	19	rs587776577	ND
Neph33	*WT1*	c.1178 G > A	p.Cys393Tyr	AD	de novo	novel	4	6	—	ND
Neph48	*WT1*	c.1491 T > A	p.Asp497Glu	AD	ND	novel	175	113	—	ND
Neph52	*WT1*	c.1432 + 5 G > A		AD	ND	reported	63	50	rs587776576	ND
Neph90	*WT1*	c.1392 C > A	p.Asp464Glu	AD	de novo	reported	5	8	—	ND
Neph92	*WT1*	c.1300 C > T	p.Arg434Cys	AD	ND (father)	reported	36	42	rs121907910	ND
Neph95	*WT1*	c.1384 C > T	p.Arg462Trp	AD	ND	reported	5	8	rs121907900	ND
Neph107	*WT1*	c.1301 G > A	p.Arg434His	AD	de novo	reported	1	3	rs121907901	ND
Neph132	*WT1*	c.1301 G > T	p.Arg434Leu	AD	de novo	reported	163	129	—	ND
Neph136	*WT1*	c.1432 + 4 C > T		AD	ND	reported	74	46	rs587776577	ND
Neph137	*WT1*	c.1432 + 4 C > T		AD	ND (father)	reported	45	45	rs587776577	ND
Neph154	*WT1*	c.1321 C > T	p.His441Tyr	AD	de novo	reported	257	216	—	ND
Neph171	*WT1*	c.1348 C > T	p.Pro450Ser	AD	de novo	reported	99	89	—	ND
Neph185	*WT1*	c.1432 + 4 C > T		AD	ND (father)	reported	48	50	rs587776577	ND
Neph197	*WT1*	c.1351 T > A	p.Phe451Ile	AD	de novo	novel	20	22	—	ND
Neph208	*WT1*	c.1334 A > G	p.His445Arg	AD	de novo	reported	46	56	—	ND
Neph27	*NPHS1* (NM_004646.3)	c.1102 C > T c.2515del	p.Pro368Ser p.Gln839ArgfsTer8	AR	father(carrier) mother(carrier)	reported reported	50 4	46 8	386833866 386833918	ND ND
Neph69	*NPHS1*	c.869dup c.1379 G > A	p.Thr291HisfsTer51 p.Arg460Gln	AR	ND	novel reported	224 274	240 199	— rs386833880	ND ND
Neph91	*NPHS1*	c.2515del c.105 G > C	p.Gln839ArgfsTer8 p.Trp35Cys	AR	father(carrier) mother(carrier)	reported novel	29 20	14 28	rs386833918 —	ND ND
Neph104	*NPHS1*	c.2515del	p.Gln839ArgfsTer8	AR	ND	reported	1	44	rs386833918	ND
Neph106	*NPHS1*	c.1135 C > T c.2515del	p.Arg379Trp p.Gln839ArgfsTer8	AR	mother(carrier) father(carrier)	reported reported	55 29	44 24	rs386833871 rs386833918	ND ND
Neph113	*NPHS1*	c.1379 G > A c.2464 G > A	p.Arg460Gln p.Val822Met	AR	mother(carrier) father(carrier)	reported reported	21 21	22 21	rs386833880 rs267606918	ND ND
Neph192	*NPHS1*	c.2464 G > A	p.Val822Met	AR	parents(carrier)	reported	31	7	rs267606918	ND
Neph203	*NPHS1*	c.3162_3165dup c.2464 G > A	p.Gly1056PhefsTer41 p.Val822Met	AR	father(carrier) mother(carrier)	reported reported	281 44	216 7	— rs267606918	ND ND
Neph59	*INF2* (NM_022489.3)	c.134 C > T	p.Pro45Leu	AD	father	novel	177	161	—	ND
Neph76	*INF2*	c.533 T > C	p.Phe178Ser	AD	mother	novel	135	160	—	ND
Neph134	*INF2*	c.529 C > T	p.Arg177Cys	AD	de novo	reported	153	172	—	ND
Neph155	*INF2*	c.218 G > T	p.Gly73Val	AD	de novo	novel	62	34	rs918089359	ND
Neph177	*INF2*	c.653 G > A	p.Arg218Gln	AD	ND	reported	103	127	rs267607183	ND
Neph198	*INF2*	c.301 T > C	p.Cys101Arg	AD	mother	novel	117	87	—	ND
Neph202	*INF2*	c.124 C > Tc.172 G > A	p.Leu42Phe p.Glu58Lys	AD	ND	novel	51 92	76 97	—	ND ND
Neph227	*INF2*	c.658 G > A	p.Glu220Lys	AD	de novo	novel	128	141	—	ND
Neph5	*TRPC6*(NM_004621.5)	c.517 T > G	p.Tyr173Asp	AD	de novo	novel	8	1	—	ND
Neph24	*TRPC6*	c.2645-1 G > A		AD	de novo	novel	82	66	—	ND
Neph39	*TRPC6*	c.2683 C > T	p.Arg895Cys	AD	de novo	reported	112	72	rs121434394	ND
Neph139	*TRPC6*	c.523 C > T	p.Arg175Trp	AD	de novo	reported	356	214	rs869025541	ND
Neph149	*TRPC6*	c.326 G > A	p.Gly109Asp	AD	de novo	novel	216	222	—	ND
Neph176	*TRPC6*	c.2624 A > T	p.Glu875Val	AD	mother	novel	225	226	—	ND
Neph234	*TRPC6*	c.434 A > G	p.His145Arg	AD	father	novel	46	113	—	ND
Neph4	*LAMB2* (NM_002292.3)	c.225del c.2095 G > C	p.Tyr76ThrfsTer36 p.Gly699Arg	AR	mother(carrier) father(carrier)	novel reported	21 32	14 26	— rs28364667	ND HGVD:0.005
Neph23	*LAMB2*	c.482 T > C	p.Leu161Pro	AR	ND	novel	0	10	—	ND
Neph58	*LAMB2*	c.4519 C > T c.1648C > T	p.Gln1507Ter p.Arg550Ter	AR	mother(carrier) father(carrier)	novel novel	239 114	195 102	rs974891221 rs1218889239	ND ND
Neph87	*LAMB2*	c.4904_4905del c.250-14_250-3del	p.Thr1635ArgfsTer23	AR	ND	novel novel	269 73	294 43	—	ND ND
Neph89	*LAMB2*	c.821 T > C	p.Leu274Pro	AR	mother(carrier)	reported	5	8	—	ND
Neph133	*LAMB2*	c.4616 G > A c.4904_4905del	p.Arg1539Gln p.Thr1635ArgfsTer23	AR	mother(carrier)	novel reported	137 178	105 152	rs758539618 —	ND ND
Neph36	*ADCK4* (NM_024876.3)	c.737 G > A	p.Ser246Asn	AR	mother(carrier) father(carrier)	novel	0	526	rs200841458	HGVD:0.002
Neph56	*ADCK4*	c.737 G > A c.532 C > T	p.Ser246Asn p.Arg178Trp	AR	ND	novel reported	287 171	281 165	rs200841458 rs398122978	HGVD:0.002 HGVD: <0.001
Neph160	*ADCK4*	c.532 C > T c.737 G > A	p.Arg178Trp p.Ser246Asn	AR	father(carrier) mother(carrier)	reported reported	163 260	173 260	rs398122978 rs200841458	HGVD: <0.001 HGVD:0.002
Neph225	*ADCK4*	c.1468 C > T c.737 G > A	p.Arg490Cys p.Ser246Asn	AR	ND	novel novel	91 159	91 159	rs750037594 rs200841458	ExAC_EAS: <0.001 HGVD:0.002
Neph19	*NUP107* (NM_020401.2)	c.1079_1083del c.2492 A > C	p.Glu360GlyfsTer6 p.Asp831Ala	AR	mother(carrier)	reported reported	1 16	1 8	— rs864321632	ND HGVD: <0.001
Neph66	*NUP107*	c.1079_1083del c.1547 A > G	p.Glu360GlyfsTer6 p.Gln516Arg	AR	father(carrier) mother(carrier)	novel novel	19 17	12 3	—	ND ND
Neph147	*NUP107*	c.2492 A > C	p.Asp831Ala	AR	mother(carrier)	reported	0	145	rs864321632	HGVD: <0.001
Neph68	*LMX1B* (NM_002316.3)	c.544 G > A	p.Asp182Asn	AD	ND	reported	316	249	rs781341216	ExAC_EAS: <0.001
Npeh77	*LMX1B*	c.737 G > A	p.Arg246Gln	AD	father	reported	7	5	rs1191455921	ND
Neph37	*ACTN4* (NM_004924.4)	c.671 T > C	p.Leu224Pro	AD	de novo	novel	213	236	—	ND
Neph146	*ACTN4*	c.912 + 1 G > A		AD	ND		120	106	—	ND
Neph129	*PAX2* (NM_003987.3)	c.71 G > C	p.Gly24Ala	AD	ND		28	51	—	ND
Neph230	*PAX2*	c.215 A > C	p.Tyr72Ser	AD	de novo	novel	58	59	—	ND
Neph178	*COL4A5* (NM_000495.4)	c.2475_2483del	p.Pro826_Gly828del	X-linked	ND	reported	127	84	—	ND
Neph204	*COL4A5*	c.438 + 5 G > A		X-linked	father	reported	208	178	rs281874739	ND
Neph97	*COQ6* (NM_182476.2)	c.782 C > T heterozygous deletion	p.Pro261Leu	AR	mother(carrier) father(carrier)	reported	24	12	rs371260604	ExAC_EAS: <0.001
Neph79	*FAT1* (NM_005245.3)	c.12867dup c.5480_5483del	p.Glu4290ArgfsTer30 p.Gly1827ValfsTer6	AR	ND	novel	179 125	135 81	—	ND ND
Neph189	*PLCE1* (NM_016341.3)	c.2674_2675dup	p.Trp893ProfsTer3	AD	parents(carrier)	novel	293	303	—	ND
Neph10	*SMARCAL1* (NM_014140.3)	c.678_679del c.2416 T > C	p.Gly227ValfsTer36 p.Trp806Arg	AR	mother(carrier) father(carrier)	novel novel	33 79	26 77	— —	ND ND
Neph143	*TTC21B* (NM_024753.4)	c.3225_3226insTGTCAAAG c.379 G > A	p.Gly1076CysfsTer29 p.Ala127Thr	AR	mother(carrier) father(carrier)	novel reported	19 158	11 148	— rs769548518	ND HGVD: <0.001
Neph216	*MYH9* (NM_002473.5)	c.2441 G > A	p.Arg814Gln	AD	mother	novel	69	79	rs760924443	ExAC_EAS: <0.001
Neph224	*CUBN* (NM_001081.3)	c.10245 C > A c.5733 + 1 G > T	p.Tyr3415Ter	AR	father(carrier) mother(carrier)	novel novel	151 144	68 128	—	ND ND
Neph236	*LAMA5* (NM_005560)	c.9232 C > T c.1282 + 1 G > A	p.Arg3078Ter	AR	father(carrier) mother(carrier)	novel novel	172 160	170 138	rs369268267 rs1168208619	ND ND

Abbreviations: AD, autosomal dominant; AR, autosomal recessive; EAS, East Asian; ExAC, Exome Aggregation Consortium; HGMD, Human Gene Mutation Database; HGVD, Human Genetic Variation Database.

We detected the disease-causing gene mutation in 85% of patients with CNS, 53% of patients with INS, 26% of patients with onset age of 1–3 years, 17% of patients with onset age of 4–6 years, 31% of patients with onset age of 7–12 years, 20% of patients with onset age of 13–18 years, and 20% of patients with onset age of 19 years or older (Supplementary Fig. S2). *WT1* gene variants were most common, detected in 17 patients; variants in *NPHS1* and *INF2* were detected in eight patients, variants in *TRPC6* were detected in seven patients, and variants in *LAMB2* were detected in six patients (Table 3).

**Table 3:** Genes with disease-causing mutations in 230 Japanese patients with proteinuria.

Causative Gene	N
*WT1*	17
*NPHS1*	8
*INF2*	8
*TRPC6*	7
*LAMB2*	6
*ADCK4*	4
*NUP107*	3
*LMX1B*	2
*ACTN4*	2
*PAX2*	2
*COL4A5*	2
Others(*COQ6, FAT1, PLCE1, SMARCAL1, TTC21B, MYH9, CUBN,LAMA5*)	8
causative mutations not detected	161

Genes with disease-causing mutations within the first 1 year of life were as follows: 37% of mutations in *WT1*, 26% of mutations in *NPHS1*, 26% of mutations in *LAMB2*, and 11% of mutations in other genes. *WT1* was also the most frequent gene mutated in individuals with onset of SRNS after the age of 1 year. In 27 (12%) patients, one or several extra-renal abnormalities were reported; these included symptoms suggestive of Denys-Drash syndrome (caused by *WT1* gene mutation and characterised by nephropathy, Wilms tumour, and genital abnormalities) and Pierson syndrome (caused by *LAMB2* gene mutation and characterised by the occurrence of congenital nephrotic syndrome and ocular anomalies in combination with microcoria).

### Renal prognosis

Seven patients had progressed to chronic kidney disease (CKD) stage 5 by the time of genetic analysis. The estimated glomerular filtration rate was above 90 ml/min/1.73 m^2^ in 108 of 173 evaluable patients (62%); it was 60 to 89 ml/min/1.73 m^2^ in 36 patients (21%), 30 to 59 ml/min/1.73 m^2^ in 18 patients (10%), and below 30 ml/min/1.73 m^2^ in 11 patients (6%) (Table 4). Kaplan–Meier analysis of renal survival showed that patients with genetic proteinuria exhibited faster progression to CKD stage 4 (p < 0.0221; Supplementary Fig. 3). The most common histopathologic diagnosis was FSGS (62%), followed by minor glomerular abnormalities (28%), mesangioproliferative GN (4%), and diffuse mesangial sclerosis (2%) (Table 5).

**Table 4:** Numbers of proteinuric patients stratified by renal function stage.

CKD stage	eGFR (ml/min/1.73 m^2^)	N (%)
1	≥90	108 (62)
2	60–89	36 (21)
3	30–59	18 (10)
4	15–29	8 (5)
5	<15	3 (2)

Abbreviations: CKD, chronic kidney disease; eGFR, estimated glomerular filtration rate.

**Table 5:** Histopathologic diagnoses of 230 Japanese patients with proteinuria.

Histopathologic diagnosis	N (%)
FSGS	124 (62)
MGA	55 (28)
MesPGN	7 (4)
DMS	3 (2)
Others	11 (6)

Abbreviations: FSGS, focal segmental glomerular sclerosis; MGA, minor glomerular abnormalities; MesPGN, mesangial proliferative glomerulonephritis; DMS, diffuse mesangial sclerosis.

### Clinical characteristics

When patients were stratified on the basis of causative mutation detection, patients with mutations in the analysed genes had higher frequencies of family history (p = 0.0004) and younger age (p = 0.024) than patients without mutations in the analysed genes. Patients with mutations had lower frequencies of nephrotic syndrome (p = 0.0421), oedema (p = 0.0018), and remission (p = 0.0104) than patients without mutations (Table 6).

**Table 6:** Comparison of clinical phenotype between patients with and without mutations in the analysed genes in Japanese patients.

	Patients with mutations	Patients without mutations	*p* value
Age*	3 (0.6–7)	4 (2–9)	0.024
Male, % (n)	55% (38/69)	58% (94/161)	0.6415
Extra-renal presentation, % (n)	12% (8/69)	12% (19/161)	0.9643
Family history, % (n)	23% (16/69)	6.9% (11/160)	0.0004
Not nephrotic syndrome, % (n)	46% (32/69)	32% (52/161)	0.0421
No oedema, % (n)	61% (41/67)	38% (58/151)	0.0018
No remission, % (n)	94% (65/69)	81% (128/158)	0.0104
FSGS, % (n)	63% (35/56)	62% (89/144)	0.9276

*Median (interquartile range).

Six parameters (age, sex, family history, oedema, remission, and nephrotic syndrome) were entered in multivariate logistic regression analysis. Patients with mutations had a significantly higher frequency of family history than patients without mutations (OR = 8.85; 95% CI = 2.96–26.48; p < 0.0001). Patients with mutations were approximately six-fold more likely to show absence of oedema (OR = 6.67; 95% CI = 2.03–21.84; p = 0.0017) and absence of remission (OR = 4.67; 95% CI = 1.39–15.69; p = 0.0128), relative to patients without mutations. Patients with mutations had higher odds of younger age than patients without mutations (OR = 0.89; 95% CI = 0.82–0.95; p < 0.0001) (Table 7).

**Table 7:** Multivariate logistic regression analysis of risk factors for patients with mutations.

95% CI	Risk factor	Odds Ratio	P value
0.82–0.95	Age	0.89	<0.0001
0.62–2.31	Sex(Male)	1.2	0.5944
2.96–26.48	Family History	8.85	<0.0001
2.03–21.84	No oedema	6.67	0.0017
1.39–15.69	No remission	4.67	0.0128
0.83–8.83	Not nephrotic syndrome	2.71	0.0872

## Discussion

In this study, we found that 30% (69 of 230) of the patients had a single gene defect in one of 60 currently known podocyte-related genes in the Japanese population. In a previous study^8^, genetic diagnoses were established in 526 patients from 183 families (detection rate of 29.5%); four genes were identified as major SRNS genes: *NPHS2* (9.93%), *NPHS1* (7.34%), *WT1* (4.77%), and *PLCE1* (2.17%). The highest rate of mutation detection (69.4%) was recorded in the youngest group of patients (0–3 months); this proportion decreased with age. In the PodoNet study^15^, genetic disease was identified in 23.6% of patients; the most common mutated genes were *NPHS2*, *WT1*, and *NPHS1*. In that report, the proportion of patients with gene mutations also decreased with age; it was 66% in patients with CNS, whereas it decreased to 15–16% in older children. In the PodoNet study, the distribution of causative genes in patients with CNS was as follows: 40% had mutations in *NPHS1*, 10.6% had mutations in *NPHS2*, 8.5% had mutations in *WT1*, 5.5% had mutations in *LAMB2*, and 4.7% had mutations in all other genes (combined). In the present study, genetic diagnoses were established in 69 of 230 unrelated patients (30%); the mutation detection rate was similar. Furthermore, common genes were *WT1* (25%), *NPHS1* (12%), *INF2* (12%), *TRPC6* (10%), and *LAMB2* (9%). In the present study, the distribution of causative genes in patients with CNS was as follows: 36% had mutations in *NPHS1*, 36% had mutations in *LAMB2*, and 18% had mutations in *WT1*. No *NPHS2* mutations were detected in our study, which was consistent with the results of a study of Korean children with SRNS (patients with CNS were excluded from that study)^16^. In China, the most common mutated genes were *ADCK4* (6.67%), *NPHS1* (5.83%), *WT1* (5.83%), and *NPHS2* (3.33%)^9^. The results of these studies show that there are differences in the types and frequencies of mutations among ethnicities and regions.

The Child Welfare Law, passed in 1961 in Japan, mandated urinary screening for preschool children, typically at 3 years of age. The purpose of urinary screening for preschool children was to prevent progression to ESRD or to improve the quality of life of children who were expected to develop ESRD. This first urinalysis is performed by using dip-and-reagent strips. In our study, we detected the disease-causing gene mutation in 41% of patients at the age of 3 years; this high detection rate was likely because of the mandatory urine screening for preschool children, which helped to detect the presence of proteinuria and could increase the likelihood that genetic analyses were conducted in affected children.

The treatment of SRNS is a challenging task for nephrologists because of its poor response to immunosuppressive drugs. High-dose steroids, cyclophosphamide, calcineurin inhibitors, mycophenolate mofetil, and rituximab have been used with variable success rates in children. However, complete remission of non-genetic SRNS was observed in 78% of patients during calcineurin inhibitor therapy^17^. In contrast, genetic SRNS was associated with a high rate of ESRD development: one patient with genetic SRNS experienced complete remission and 16% of patients with genetic SRNS experienced partial remission after calcineurin inhibitor therapy^17^. In our study, complete remission of SRNS without mutations was observed in 26% of patients during immunosuppressive therapy. However, this proportion does not reflect the natural clinical course of SRNS because most patients with SRNS who do not have mutations will be treated with immunosuppressants, such as repeated steroid pulses or rituximab treatment after genetic analyses, and there is insufficient long-term follow-up data for these types of patients. Complete remission of nephrotic syndrome in patients with mutations was observed in 5% (2/37) of patients during treatment with immunosuppressive therapies and in one patient during treatment with angiotensin-converting enzyme inhibitors. Notably, angiotensin-converting enzyme inhibitors and angiotensin receptor blockers may cause urinary protein reduction and have renoprotective effects. However, a previous study showed that more patients reached ESRD when using calcineurin inhibitors; the investigators concluded that calcineurin inhibitors may cause proteinuria reduction, but may negatively influence kidney function^17^. Most patients with nephrotic syndrome who have mutations do not respond to immunosuppressive therapy; however, patients with disease-causing mutations in *PLCE1*^18^ and *TRPC6*^19^ at least partially respond to therapy. Thus far, there are insufficient data to determine whether this proteinuria reduction has a renoprotective effect, and further studies of extended cohorts are needed.

The identification of gene mutations is important for decision-making in terms of future treatment strategies and predicting prognosis. However, it is not yet feasible to perform genetic testing in all patients with proteinuria. Therefore, it is necessary to consider which patients should undergo genetic testing. In previous reports of patients with SRNS, the likelihood of identifying a genetic mutation was inversely related to age at disease onset and was increased in patients with positive family history and in those with extra-renal manifestations^7^. In our study, we compared patients with and without known causative mutations, among all patients. The results showed that risk factors of genetic disease were younger age, family history, absence of oedema, and absence of remission. Importantly, absence of remission was strongly associated with genetic disease in this study. In a previous report of adult-onset FSGS, secondary FSGS (i.e., resistance to immunosuppression and atypical primary FSGS) was considered for genetic evaluation^20^; our results concur with those of the prior report.

This study had several limitations. First, this was a cross-sectional retrospective study with a small study population, which limits the generalisability of the findings. Second, the examinations of individual patients relied entirely on the attending clinicians’ decisions, due to the retrospective nature of the study. Finally, treatment details were not accessible for some patients, which limited our ability to make inferences regarding their disease characteristics.

In conclusion, we found pathogenic disease-causing gene mutations in Japanese patients with severe proteinuria. Detection of these mutations in podocyte-related genes will be helpful in treatment and prediction of renal outcome.

## Methods

### Patients

This study protocol was approved by the Institutional Review Board of Kobe University Graduate School of Medicine (IRB approval number 301). Informed consent was obtained from the patients or their family members in this study. The patients were recruited between January 2016 and December 2018. Inclusion in the study was based on fulfilment of one of the following criteria: (i) diagnosis of CNS, which presents within the first 3 months of life; (ii) diagnosis of INS, which presents between 3–12 months of age; (iii) diagnosis of SRNS, which is defined by persistent proteinuria after 4 weeks of daily treatment with 60 mg/m^2^ prednisone; (iv) diagnosis of FSGS or asymptomatic proteinuria. Asymptomatic proteinuria was defined as the absence of who extra-renal symptoms or the presence of proteinuria and microhaematuria. (Supplementary Table S2). Details regarding family history and other clinical features were obtained from the referring clinician or the patient’s hospital records. eGFR was calculated for ages 3 month to 18 years using the creatinine based eGFR formula in Japanese child^21,22^. For the patients aged <3 month, eGFR was calculated using the original Schwartz formula^23^ as follows: *k* × body length (cm)/serum Cr level (mg/dL). For this study, the *k* values was set as 0.45.

### Genetic analysis

As we previously reported^24^, genomic DNA was isolated from peripheral blood leukocytes from patients and their family members using the Quick Gene Mini 80 system (Wako Pure Chemical Industries, Ltd., Tokyo, Japan), in accordance with the manufacturer’s instructions. Targeted sequencing using NGS was conducted for genes that are associated with inherited glomerular diseases (Supplementary Table S3). NGS samples were prepared using a HaloPlex target enrichment system kit (Agilent Technologies, Santa Clara, CA, USA), in accordance with the manufacturer’s instructions. All indexed DNA samples were amplified by polymerase chain reaction and sequenced using the MiSeq platform (Illumina, San Diego, CA, USA). Resulting sequence data were analysed (from alignment to categorisation of mutations) using SureCall software (version 4.0, Agilent Technologies). As we previously reported^24^, pair analysis by SureCall was used to determine copy number changes in experimental samples relative to a reference sample without a copy number change. We conducted an additional custom array comparative genomic hybridisation when the identified exons (more than two exons) in a single patient exhibited deletions that were all consistent with the clinical presentation of the patient.

### Custom array comparative genomic hybridisation

As we previously reported^25^, we conducted custom array comparative genomic hybridisation for one patient. We selected the *COQ6* gene and constructed probes for it and the regions surrounding it. We used a custom HD-comparative genomic hybridisation microarray, 8 × 15 K (Agilent Technologies), in accordance with the manufacturer’s instructions. We used Agilent CytoGenomics software (Agilent Technologies) to analyse chromosomal patterns within the microarray profiles.

### Confirmation of the pathogenicity

Candidate variants were considered disease-causing mutations when they met at least one of the following criteria: (1) previous identification as a disease-causing mutation in a published paper; (2) predicted truncation (i.e., nonsense, obligatory splice site, or frameshift mutations); (3) for all novel missense variants, in silico testing with MutationTaster (http://mutationtaster.org/), PolyPhen-2 (http://genetics.bwh.havard.edu/pph/), or SIFT (http://sift.jcvi.org/) indicated pathogenicity. In addition to these three criteria, we confirmed the absence of contradictions between familial segregation and symptoms.

### Statistical analysis

Results are presented as median and interquartile range (IQR). The chi-squared test or Fisher’s exact test was used to compare variables between two groups. The Mann-Whitney U test was used to compare median differences between two experimental groups. Multivariate logistic regression analysis was performed to calculate odds ratios (ORs) and 95% confidence intervals (95% CIs) after controlling for potential confounders. Statistical analysis was performed using standard statistical software (JMP version 10 for Windows; SAS Institute, Cary, NC, USA). In all tests, p < 0.05 was considered statistically significant.

## Supplementary information

Supporting Information.

## Data Availability

The data are not available for public access because of patient privacy concerns, but are available from the corresponding author on reasonable request.
