# Supplementary material for: Comprehensive genetic diagnosis of Japanese patients with severe proteinuria
Source: Sci Rep. 2020 Jan 14;10:270. doi: 10.1038/s41598-019-57149-5 (PMC6959278; doi:10.1038/s41598-019-57149-5)
Supplement: Supplementary file 1 — Supporting Information. [file 41598_2019_57149_MOESM1_ESM.pdf]

Supplementary data

Title:

Comprehensive genetic diagnosis of Japanese patients with severe proteinuria

China Nagano, Tomohiko Yamamura, Tomoko Horinouchi, Yuya Aoto, Shinya Ishiko, Nana Sakakibara, Yuko Shima, Koichi Nakanishi, Hiroaki Nagase, Kazumoto Iijima, Kandai Nozu

Supplementary data

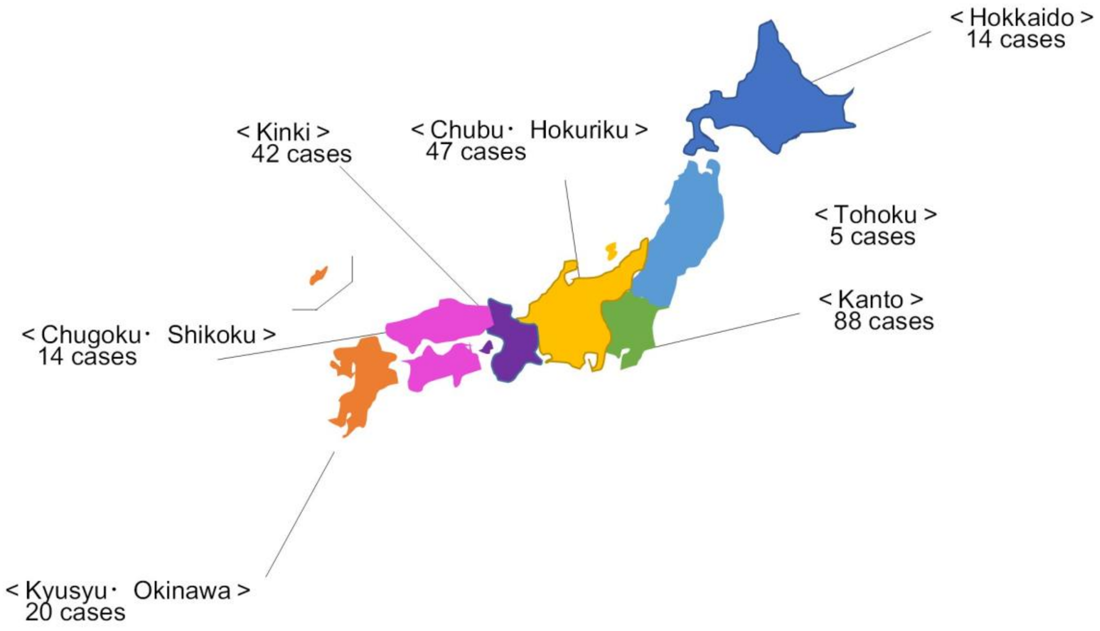

Supplementary Fig. S1 Map of regions where samples were collected. A total of 230 samples were collected from 89 institutions. Figure was produced by Microsoft PowerPoint 2013 (https://products.office.com/en-us/powerpoint).

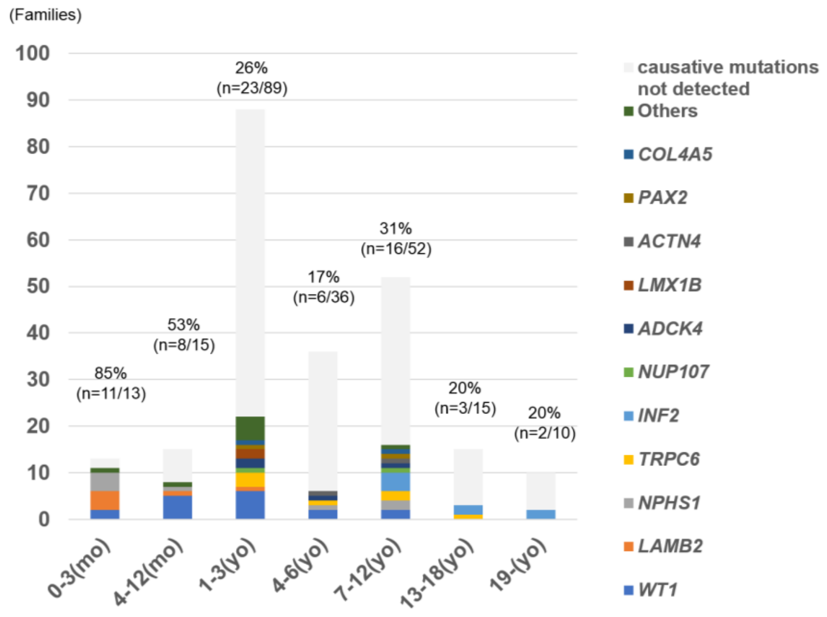

Supplementary Fig. S2 Histograms show proportions of patients who had corresponding disease-causing gene mutations in each age group

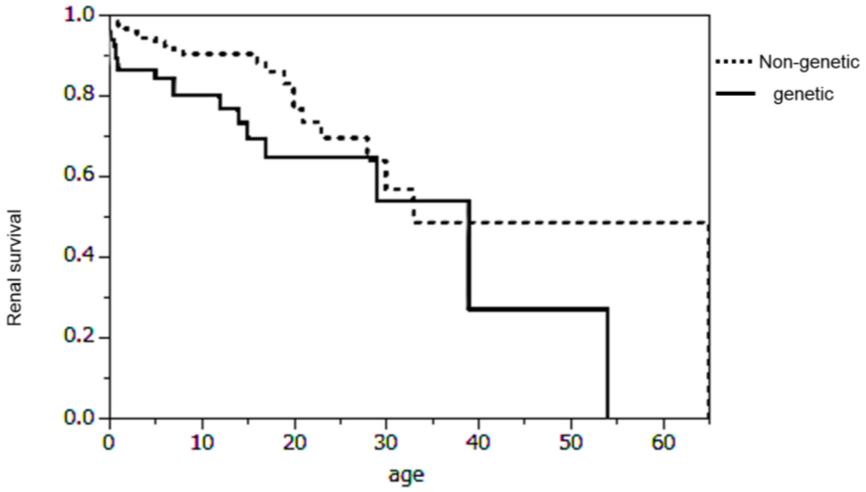

Supplementary Fig. S3 Kaplan–Meier survival analysis of the progression of genetic and non-genetic chronic kidney disease (CKD) with the end-point at stage 4 CKD (P = 0.0221). Figure was produced by JMP 10 (SAS Institute Inc., Cary, NC, USA).

Supplementary Table S1：Targeted gene coverages

Gene	Regions	Coverage	High Coverage (≧90%)	Low Coverage (<90%)
WT1	11	100%	11	0
NPHS1	29	100%	29	0
INF2	22	100%	22	0
TRPC6	13	100%	13	0
LAMB2	32	100%	32	0
ADCK4	14	100%	14	0
NUP107	28	100%	28	0
LMX1B	8	100%	8	0
ACTN4	25	99.27177%	24	1
PAX2	13	100%	13	0
COL4A5	55	99.53488%	54	1
COQ6	13	100%	13	0
FAT1	29	100%	29	0
PLCE1	33	99.32776%	32	1
SMARCAL1	17	100%	17	0
TTC21B	29	100%	29	0
MYH9	41	100%	41	0
CUBN	69	100%	69	0
LAMA5	83	98.6424%	81	2

Supplementary Table S2. Clinical features of patients with asymptomatic proteinuria

Patient	Gene	genome	amino acids	Gender	Age at onset(y)	ESRD(y)	Histopathologic diagnosis	Family history**	Extrarenal symptom	Age at gene analysis(y)	eGFR(ml/min/1.73m2)
Neph11				F	6	-	MGA	-	-	8	281.2
Neph15	*WT1*<br>(NM_024426.4)	c.1432+4C>T		F	10	-	MGA	-	-	16	114.4
Neph17				F	4	-	MGA	-	-	11	140.8
Neph33	*WT1*	c.1491T>A	p.Asp497Glu	F	3	-	MGA	-	-	9	113.5
Neph51				M	34	-	MGA	1	-	44	110.5
Neph59	*INF2*<br>(NM_022489.3)	c.134C>T	p.Pro45Leu	F	13	-	Focal glomerular obsolescent	2	-	25	113
Neph66	*NUP107*<br>(NM_020401.2)	c.1079_1083del<br>c.1547A>G	p.Glu360GlyfsTer6<br>p.Gln516Arg	F	3	-		2	-	3	129.4
Neph70	*WT1*	c.1384C>T	p.Arg462Trp	F	27	-	MGA	2	-	27	105
Neph79	*FAT1*<br>(NM_005245.3)	c.12867dup<br>c.5480_5483del	p.Glu4290ArgfsTer30<br>p.Gly1827ValfsTer6	M	3	-	MGA	-	-	5	141.8
Neph80				F	6	-	MGA	-	-	17	133.9
Neph94				F	17	-		-	-	37	71.2
Neph96				F	8	-	MGA	-	-	13	72.5
Neph99				F	3	-	MGA	-	-	8	155.7
Neph100				M	6	-	global sclerosis	-	-	6	25
Neph103				F	3	-	MGA	-	-	14	130.9
Neph128				M	3	-	MGA	-	-	4	100.13
Neph136	*WT1*	c.1432+4C>T		F	6	-	MGA	-	-	17	83.4
Neph152				M	3	-	MGA	-	-	7	174.3
Neph167				M	3	-	MGA	-	-	22	217
Neph184				F	3	-	MGA	-	-	11	125.1
Neph190				M	3	-	MGA	2	-	4	59.9
Neph195				F	3	-	MGA	1	-	4	121.8
Neph198	*INF2*	c.301T>C	p.Cys101Arg	F	13	-	MGA	2	-	16	115.2
Neph204	*COL4A5*<br>(NM_000495.4)	c.438+5G>A		F	1	-	non-IgA mesangial proliferateve GN	2	-	17	93.3
Neph222				M	2	-	MGA	-	-	7	105.4
Neph224	*CUBN*<br>(NM_001081.3)	c.10245C>A<br>c.5733+1G>T	p.Tyr3415Ter	M	3	-	MGA	-	-	3	112

**1: positive family history of proteinuria, 2: positive family history of renal failure.
ESRD: End-stage renal disease, FSGS: Focal segmental glomerular sclerosis, MGA: Minor glomerular abnormality, GN: Glomerular nephritis

Supplementary Table S3. List of 60 podocyte-related genes included in targeted sequencing analysis within a clinically approved gene panel test developed in our laboratory.

ACTN4	*COQ2*	*ITGB4*	*MYH9*	*SCARB2*
ADCK4	*COQ6*	*ITSN1*	*MYO1E*	*SGPL1*
ANKFY1	*CRB2*	*ITSN2*	*NPHS1*	*SMARCAL1*
ANLN	*CUBN*	*KANK1*	*NPHS2*	*TNS2*
ARHGAP24	*DGKE*	*KANK2*	*NUP107*	*TP53RK*
ARHGDIA	*DLC1*	*KANK4*	*NUP205*	*TPRKB*
AVIL	*EMP2*	*LAGE3*	*NUP93*	*TRPC6*
CD2AP	*FAT1*	*LAMA5*	*OSGEP*	*TTC21B*
CDK20	*GAPVD1*	*LAMB2*	*PAX2*	*WDR4*
COL4A3	*PTPRO*	*LMNA*	*PDSS2*	*WDR73*
COL4A4	*INF2*	*LMX1B*	*PLCE1*	*WT1*
COL4A5	*ITGA3*	*MAGI2*	*PTPRO*	*XPO5*
